# The role of parents in behavioral treatment for adolescent obesity: design and rationale for the TEENS+ randomized clinical trial

**DOI:** 10.1186/s12889-023-16421-0

**Published:** 2023-08-04

**Authors:** Melanie K. Bean, Jessica Gokee LaRose, Edmond P. Wickham, Hollie A. Raynor, Laura Caccavale, Ronald K. Evans, Laura M. Thornton, Sarah Farthing, Ashley Mendoza, Suzanne E. Mazzeo

**Affiliations:** 1 Department of Pediatrics, School of Medicine, Children’s Hospital of Richmond at Virginia Commonwealth University, Box 980140, Richmond, VA 23298 USA; 2 Department of Psychiatry, School of Medicine, Virginia Commonwealth University, Box 980308, Richmond, VA 23298 USA; 3 Department of Health Behavior and Policy, School of Medicine, Virginia Commonwealth University, Box 980430, Richmond, VA 23298 USA; 4 Department of Internal Medicine, School of Medicine, Virginia Commonwealth University, Box 980111, Richmond, VA 23298 USA; 5 Department of Nutrition, University of Tennessee, Knoxville, 1215 W. Cumberland Ave., Knoxville, TN 37996 USA; 6 Department of Kinesiology and Health Sciences, College of Humanities and Sciences, Virginia Commonwealth University, Box 843021, Richmond, VA 23284 USA; 7 Department of Psychiatry, School of Medicine, University of North Carolina at Chapel Hill, CB#7160 101 Manning Drive, Chapel Hill, NC 27599-7160 USA; 8 Department of Psychology, College of Humanities and Sciences, Virginia Commonwealth University, Box 842018, Richmond, VA 23284 USA

**Keywords:** Adolescent, Obesity, Parent, Randomized clinical trial

## Abstract

**Background:**

There is an urgent need for innovative approaches to adolescent obesity treatment, particularly among individuals from racially and ethnically marginalized backgrounds, who face increased risk of obesity and its associated morbidity and mortality. There is a particular dearth of research on the long-term efficacy of adolescent obesity treatments. Further, research and clinical practice guidelines consistently recommend parents’ inclusion in their adolescents’ obesity treatment, yet the most effective strategy to engage parents in adolescent obesity treatment remains unclear. Towards that end, this investigation will conduct a fully-powered, randomized clinical trial to examine the efficacy of two distinct approaches to involving parents in their adolescents’ obesity treatment.

**Methods:**

Participants will be 210 12-16 year old adolescents (body mass index [BMI]≥85^th^ percentile) and parents (BMI≥25 kg/m^2^) with overweight or obesity. Dyads will be randomized to one of two 4-month treatments: 1) TEENS+Parents as Coaches (PAC), engaging parents as helpers in their child’s weight management via parent skills training based on authoritative parenting, or 2) TEENS+Parent Weight Loss (PWL), engaging parents in their own behavioral weight management. All adolescents will participate in the TEENS+ protocol, which includes nutrition education with dietary goals, supervised physical activity, and behavioral support, and integrates motivational interviewing to enhance treatment engagement. Assessments of anthropometrics, dietary intake, physical activity, parenting and home environment variables will be completed at 0, 2, 4, 8, and 12 months with the primary endpoint at 12-month follow-up.

**Discussion:**

Results of this investigation have the potential to significantly advance science in this area and ultimately inform clinical practice guidelines related to the role of parents in adolescent obesity treatment.

**Trial registration:**

Clinicaltrials.gov NCT03851796. Registered: February 22, 2019.

## Background and rationale

More than one-fifth of U.S. adolescents (age 12-19 years) have obesity (body mass index [BMI]≥95^th^percentile) [1]. Prevalence is even higher among adolescents from racially and ethnically marginalized backgrounds [2]. With a looming epidemic of premature diabetes and cardiovascular disease [3–6], the need for obesity treatment targeting racially diverse adolescents is urgent. Despite this established need, relatively few randomized clinical trials (RCTs) target adolescents, and even fewer include racially and economically diverse samples [7–9]. Clinical treatment guidelines recommend family-based, lifestyle obesity interventions [10–13] and consistently emphasize the importance of including parents [10, 14]. This treatment approach is associated with modest improvements in BMI and metabolic risk factors [15–17]. However, the most effective way to engage parents in adolescent obesity treatment remains unclear, and there is limited evidence to support specific strategies for parental involvement in adolescent treatment [18], with a particular absence of evidence among ethnically/racially marginalized groups [19–21]. Moreover, very few adolescent behavioral weight loss (BWL) trials have examined maintenance effects beyond 6-months post-treatment [8, 22]. Thus, rigorous trials to examine the optimal role of parents in adolescent obesity treatment are needed.

Involving parents in family-based obesity treatment can be particularly challenging during adolescence, given normative developmental factors, such as opposition to authority, role transformations, and increasing personal responsibility [23, 24]. Yet, despite an increased drive for independence and autonomy, and the elevated influence of peers, adolescents rely on parents for many (instrumental [e.g., meals] [25] and relational [e.g., attachment] [26]) needs. Consideration of these developmental processes and data addressing best clinical practices for this age group have informed our behavioral obesity treatment approach for adolescents. Specifically, our team has developed TEENS+ (**T**eaching, **E**xercise, **E**ncouragement, **N**utrition, **S**upport) based on extensive experience conducting BWL treatment with racially and economically diverse adolescents with obesity [15, 27–29]. The current trial is based on a pilot of TEENS+, in which we randomized parents to one of two distinct interventions to determine the feasibility and acceptability of these approaches, and their potential impact on adolescent weight loss [27].

A specific focus on parents is a vital component of pediatric obesity treatment for several reasons. Specifically, parental obesity is associated with overweight in children [30]; parents are powerful role models of eating and exercise behaviors [31, 32]; parents’ feeding behaviors influence children’s eating habits and weight [33–36]; and the home environment strongly influences children’s lifestyle choices [37, 38]. Indeed, parental involvement in pediatric obesity treatment is associated with better child outcomes [12, 39]. However, there is no consensus regarding how to optimize this involvement across developmental stages [12–14, 39–41]. Available data lack specificity regarding the ideal amount and type of parental involvement and the relation between parent variables and adolescents’ BMI outcomes [7].

Parent weight change during adolescent BWL treatment is an important predictor of adolescent outcomes. For example, change in parent BMI during adolescent BWL treatment was associated with change in adolescent BMI [42]. Moreover, parents who lost more weight during a 4-month treatment had adolescents with greater % weight loss at 12-month follow-up, suggesting that engaging parents in weight loss efforts might facilitate their adolescents’ weight loss maintenance [42]. In a related trial, parent BMI change was the only independent predictor of adolescent BMI change at the end of the 4month treatment [43]. However, parents’ weight change during treatment was *not*associated with adolescents’ weight loss maintenance assessed 2 years later [9]. Importantly, parent weight loss was *not* directly targeted in any of these prior trials. Directly targeting parent weight loss could contribute to greater adolescent change in BMI, as parents engaged in concurrent weight management might make greater alterations to the shared environment and serve as particularly powerful role models.

Previous adolescent obesity treatments involving parents [28, 44] typically build parents’ skills and self-efficacy to support their adolescents’ weight management via role modeling, reinforcement of specific health behaviors, use of authoritative parenting and feeding styles, and changes in the home environment [20]. Although effects vary, trials implementing this approach, including the TEENS+ pilot [27], generally report significant adolescent BMI reductions [28, 42–44].

More specifically, in the TEENS+ pilot, we randomized parents to PAC (Parents as Coaches; parent skills training based on authoritative parenting) or PWL (Parent Weight Loss; parents engaged in their own BWL), and found significant reductions in adolescent BMI in both groups [27]. Thus, both parent involvement approaches appear viable. However, differential weight patterns began to emerge during a 3-month maintenance period, suggesting the potential for PAC to confer better weight loss maintenance. Findings suggest the potential benefit of a parent skills training approach designed to increase parents' use of authoritative parenting (as observed in PAC and not PWL), to create an emotional climate that fosters sustained weight losses in adolescents. There is thus a need to identify mechanisms of action through which parent variables impact adolescent weight loss and maintenance.

Previous trials have identified parent variables that might serve as mechanisms influencing adolescent weight loss. Specifically, authoritative parenting, characterized as being both responsive (attuned and supportive to foster autonomy) and demanding (providing structure to cultivate responsibility), is associated with healthy weight outcomes in adolescents (although conclusions are limited by a lack of longitudinal research) [35, 45]. Authoritative feeding (e.g., providing choice within parameters; avoiding excessive restriction or control) is a separate (and not necessarily related [45]) construct that is also associated with improved weight outcomes in children [12, 35, 46]. These links are evident among adolescents as well [45]. For example, greater baseline parental pressure to eat (inconsistent with authoritative feeding) was associated with lower adolescent weight loss in a BWL trial [43]. However, most extant research is cross-sectional, and thus, causality cannot be determined [45]. There is potential that the effect of these parenting variables differ among adolescents with eating pathology. For example, a review by Matheson and colleagues suggested that authoritative parenting operates differently among adolescents with obesity who also have binge and/or loss of control eating (BE/LOC) [47]. Evaluation of the potential moderating role of BE/LOC will enhance understanding of parenting factors among this complex subgroup of adolescents with obesity and BE/LOC, representing 22-31% of the clinical population [48].

**Fig. 1:**
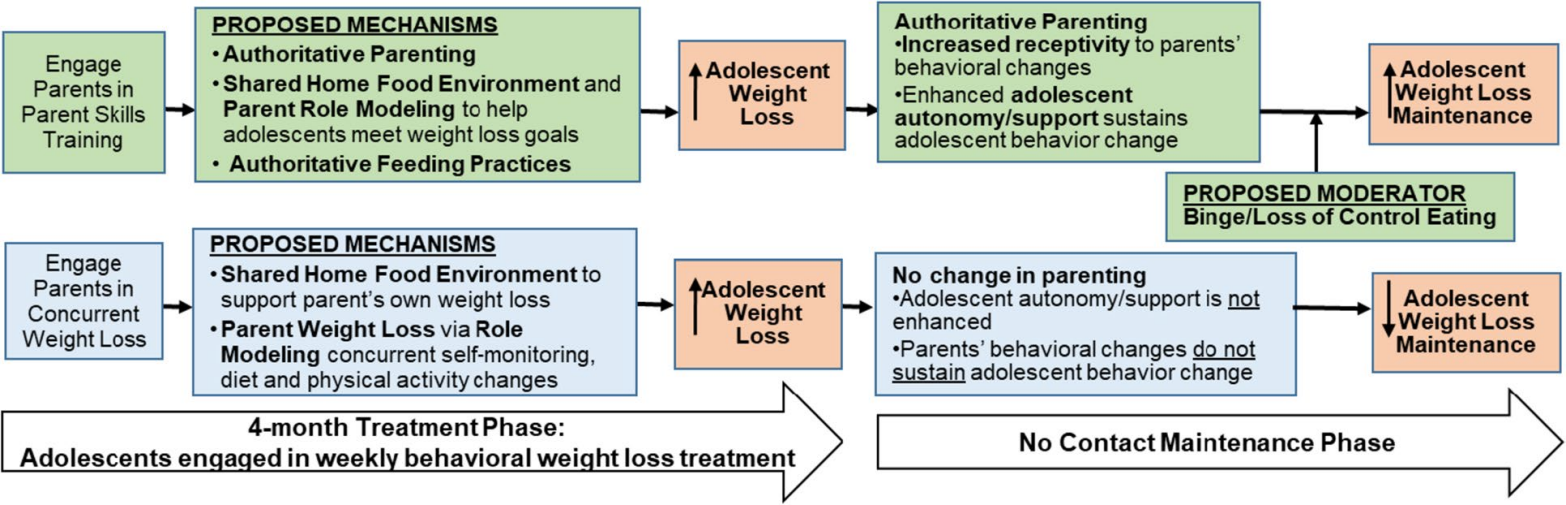
Conceptual model for parent influence on adolescent weight loss and maintenance within obesity treatment

In summary, these studies support use of a parent skills training intervention within adolescent obesity treatment, and identify authoritative parenting as a potential mechanism through which parents’ behavior influences adolescent weight loss maintenance. However, other than our pilot, no studies have directly compared parent skills training with alternative, empirically-supported parent approaches and examined their impact on adolescent outcomes. The current fully-powered trial, guided by an empirically and theoretically-supported conceptual model (Fig. 1), randomizes parents to either parent skills training based on authoritative parenting (TEENS+PAC), or to a concurrent adult BWL program (TEENS+ PWL), and examines their impact on adolescent weight loss maintenance. Thus, results will provide insight regarding: 1) the optimal approach to engaging parents in adolescent obesity treatment, and 2) key mechanisms that explain parents’ influence on adolescent weight loss maintenance. Outcomes have high potential to inform clinical practice guidelines and advance this scientific area.

### Objectives

The primary objective of this trial is to evaluate the efficacy of TEENS+PAC and TEENS+PWL on adolescent weight loss maintenance. We hypothesize that adolescents in TEENS+PAC will manifest better weight loss maintenance at 12 months (BMI change [Δ] from 4-12 months) compared with adolescents in TEENS+PWL. We also hypothesize that baseline BE/LOC will moderate the impact of treatment on adolescent weight loss maintenance: in PAC, those without BE/LOC will have better weight loss maintenance than those with BE/LOC; in PWL, there will be no difference in maintenance between those with or without BE/LOC.

A secondary aim is to evaluate mediators of the PAC and PWL treatments on adolescent weight loss maintenance. We hypothesize that parents in both groups will make significant improvements to the home food environment during the 4-month treatment. However, parents in PWL will manifest greater reductions in weight at 4 months (% weight loss) and improvements in weight management behaviors (dietary intake, physical activity, and self-monitoring) than parents in PAC. The PAC intervention will have a greater impact on parenting variables (e.g., authoritative parenting and feeding) during the 4-month treatment compared with PWL.

We further hypothesize that increases in authoritative parenting (from 0 to 4 months) will mediate the differential impact of PAC vs PWL on adolescent weight loss maintenance at 12 months (ΔBMI from 4-12 months), with baseline BE/LOC as a moderator. Specifically, increases in authoritative parenting will negatively impact weight loss maintenance among adolescents with BE/LOC and positively impact weight loss maintenance among adolescents without BE/LOC.

In addition to these primary aims, we will evaluate the efficacy of TEENS+PAC and TEENS+PWL on adolescent diet and physical activity. We hypothesize that adolescents in TEENS+PAC will achieve greater improvements in dietary intake and physical activity at 12 months compared with adolescents in TEENS+PWL.

Finally, we will explore how parent and adolescent concordance of sex, weight loss, behavioral changes (self-monitoring, dietary intake, physical activity), and perceived parenting style impacts adolescent weight loss maintenance. We will also assess how adolescents’ perceptions of their non-participating parents’ parenting style (and its concordance with their participating parent) impacts adolescent weight loss maintenance.

## Methods/design

**Fig. 2:**
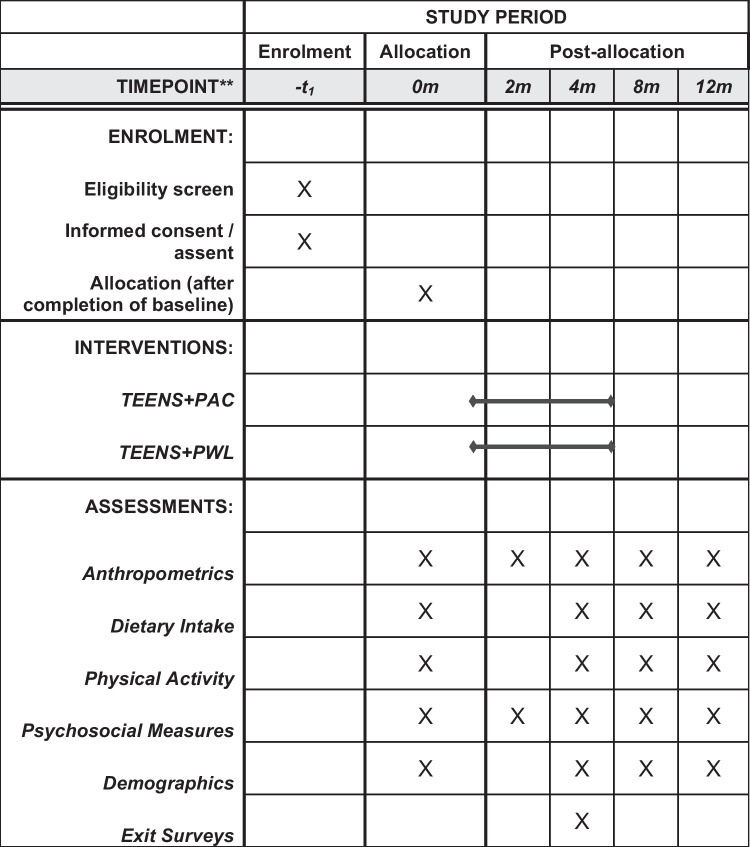
SPIRIT schedule of enrolment, intervention, and assessments for the TEENS+ randomized clinical trial

This is a 2x5 repeated-measures, RCT design. Families are randomly assigned to either PAC or PWL. All adolescents participate in the 4-month TEENS+ protocol. This trial is modeled after our pilot and designed to test its efficacy in a fully powered trial over 1-year follow-up. Assessments are conducted at 0, 2, 4, 8 and 12 months. The primary dependent variable of interest is adolescent ΔBMI at 12 months (ΔBMI_4-12m_). Following completion of baseline assessments, cohorts of adolescent/parent dyads are stratified by sex and race and randomized to TEENS+PAC or TEENS+PWL. See Fig. 2.

### Study setting

The trial was initially designed to be conducted completely in-person at Virginia Commonwealth University’s (VCU’s) Healthy Lifestyles Center (HLC). However, the COVID-19 crisis led to a transition to fully virtual treatment (via the Zoom platform) in March 2020, due to in-person research restrictions. This shift occurred during cohort 2 and the online format will be maintained for the remainder of data collection. Details of the online implementation and assessments are discussed below.

### Eligibility criteria

Adolescents are eligible if they are 12-16 years of age, have a BMI ≥85^th^ percentile for age and sex and primarily reside in the participating caregiver’s home. Parents must have overweight or obesity (BMI≥25kg/m^2^). Adolescents and parents are ineligible if they have: 1) a medical condition that might be associated with unintentional weight change; 2) a history of weight loss surgery; 3) a recent (past 3-months) change in medications that can impact weight; 4) psychiatric, medical, cognitive or developmental conditions that would impair their ability to complete assessments, exercise, or participate in a group; 5) active anorexia or bulimia nervosa; or 6) pregnancy or plan to become pregnant. All interventionists are trained health coaches (psychology doctoral students, social workers, or similar) paired with a registered dietitian. Interventionists complete manualized training and attend weekly supervision.

### Interventions

#### Theoretical foundations

Both PAC and PWL are grounded in Social Cognitive Theory, which emphasizes interactions among environmental, personal and behavioral factors and capitalizes on social learning [49–51]. Core evidence-based strategies that form the basis of BWL interventions [11, 40] are integrated into all sessions. Our interventions are further enhanced by the use of Motivational Interviewing (MI), via evoking participants’ reasons for change, highlighting autonomy, and building self-efficacy [52, 53]. Importantly, the parent treatments have distinct theoretically-informed foci: PAC focuses on parenting strategies to support child weight loss efforts; PWL uses a behavioral approach focusing on parents’ own weight loss (See Table 1). Intervention methods were piloted as described previously [27, 54] and also described below.

**Table 1:** Comparison of Parents as Coaches (PAC) with Parents Weight Loss (PWL) treatments

	**Parents as Coaches**	**Parent Weight Loss**
**Treatment Structure/ Content**	• 16-weeks; 60 min group sessions • led by trained behavioral coaches • 8 behavioral parent skills training lessons & 8 nutrition education sessions (combined with teens)	• 16-weeks; 60 min group sessions • led by trained behavioral coaches • 16 behavioral weight loss lessons (including 8 nutrition education sessions)
**Treatment Focus**	• adolescent weight management via parent skills training based on authoritative parenting approach	• parent weight loss via lifestyle intervention focused on modifications to diet, physical activity, and behavior
**Dietary Approach**	• virtual grocery store teaches label-reading and healthy shopping on a budget • emphasizes realistic strategies for family and home environment change to support child’s efforts • taught how to help their adolescent meet dietary goals via meal planning, preparing meals at home, and home environment changes • problem-solving around challenges to adherence (e.g., schedules, food preferences, cost, and stress)	• assigned diet goals based on predicted Resting Metabolic Rate (e.g., 1200-1800 kcal, 30% kcal from fat; adjusted as needed based on progress) • weekly weights obtained and adjustments made to address rapid, unsafe weight loss if needed • problem-solving around challenges to adherence (e.g., access, cost, stress, and social influences) • taught to make adjustments to dietary intake to maintain weight loss after the program
**Physical Activity (PA) Approach**	• parents learn practical (free and low-cost) strategies to increase family PA • behavioral goals focus on increasing family PA and reducing / setting limits on screen time • psychoeducation provided / problem-solving conducted about overcoming family barriers to PA	• parents instructed to increase PA until achieving ≥250 minutes/week of moderate intensity PA • multiple short bouts of PA encouraged (e.g., ≥10 min) to minimize potential barriers to activity • psychoeducation and problem-solving around challenges to PA (e.g., time, motivation, access)
**Self-Monitoring, Reporting & Feedback**	• parents keep logs of individualized parenting goals set in treatment and self-monitor progress • logs are reviewed, facilitating group-brainstorming of barriers encountered and possible solutions • parents review teen logs and assist as needed • parents meet briefly with coach individually before meeting facilitating personalized feedback	• parents keep personal logs including detailed food records and minutes / type of PA • coaches review logs & provide weekly feedback • parents taught to self-weigh at least weekly (≥4 days / week recommended) • parents weigh in before each meeting, facilitating personalized feedback / brainstorming of barriers
**Core Behavioral Strategies**	• parents taught core behavioral / parenting strategies to help implement **family changes** and support their child’s weight management	• parents taught core behavioral and cognitive skills to help implement **personal** changes for weight loss

#### Tailoring for inclusivity

Our interventions were designed with particular sensitivity to families from diverse racial and socioeconomic backgrounds [55]. This includes healthy, budget-conscious modifications to traditional meals, acknowledging cultural differences in body image ideals, and recognizing extended kinship networks. We also acknowledge unique pragmatic challenges facing low-income and single-parent families by focusing on realistic, practical behavioral strategies, emphasizing accessible and free activity options, teaching healthy and budget conscious grocery store strategies, and recognizing relevant policies (e.g., free school meals) that might impact dietary intake (e.g., teaching strategies to make healthier choices at school vs. encouraging all participants to pack a lunch, which might not be economically feasible). This emphasis was enhanced during the COVID-19 pandemic, given dramatic increases in financial instability and food insecurity [56, 57]. Finally, personnel are trained in culturally sensitive intervention delivery [58, 59]. PAC acknowledges cultural differences in parenting and feeding, yet highlights the value of an authoritative approach to both [60–62]. Parents are encouraged to make modifications to their current eating practices that fit with their cultural values and context.

#### TEENS+ adolescent intervention

To reduce potential contamination, adolescents are assigned to groups according to their parents’ treatment. Adolescent groups are single-gender (self-identified), to facilitate interaction at this critical developmental stage, led by two trained, supervised lifestyle coaches masked to study hypotheses. Over 16 weeks, adolescents meet weekly for 1-hour groups. Content in TEENS+PAC and TEENS+PWL is identical; however, in PAC, adolescents alternate weekly between concurrent, separate group visits and conjoint (parent and child) group nutrition education visits. In PWL, all core adolescent groups occur separately from their parents. TEENS+ considers the adolescent developmental tasks of individuation and autonomy development, and includes strategies to navigate peer influences, while also eliciting adolescent-driven reasons for change. TEENS+ recognizes that responsibility for eating choices shifts from parents to adolescents, yet acknowledges that parent influence remains strong (e.g., ~50% of adolescents eat ≥5 meals/week with their family) [25]. As such, autonomy is emphasized, although adolescents are encouraged to consider what they need from significant others (including parents) for successful goal attainment (e.g., asking parents to keep certain foods in/out of the home; transportation to the YMCA).

Manualized sessions follow a behavior therapy approach, including guided goal-setting, self-monitoring, identifying barriers and solutions, contingency management, stimulus control, dealing with setbacks, maintenance and relapse prevention. Weight is assessed weekly by coaches, and adolescents maintain food and physical activity logs. These logs are reviewed weekly by coaches, with personalized, written feedback provided. This feedback is contextualized within a self-regulation framework [63] that teaches adolescents to identify how their eating and exercise behaviors relate to weight change, and to adjust behaviors as needed to reach their weight management goals. A point system (1 raffle ticket/point) is implemented to reinforce attendance and log completion, consistent with behavior theory, with raffles drawn on a variable reinforcement schedule. Group incentives (e.g., water bottles) are provided monthly. In these ways, TEENS+ facilitates group cohesion and reinforces adherence and attendance, increasing engagement. Families also attend a cooking class and receive YMCA memberships. During the maintenance phase (4-12 months), monthly, arm-specific newsletters are mailed to participants to reinforce content and maintain engagement with the trial.

Adolescents also complete four 30-min individual visits. In month 1, adolescents meet with their behavior coach for an MI-informed values exploration exercise to explore the consistency of TEENS+ with adolescents’ goals and values. The purpose of this meeting is to increase adolescent’s internal motivation for change and treatment engagement. Use of MI Is consistent with prior research demonstrating its value in increasing obesity treatment engagement [64, 65]. In months 2 and 3, adolescents meet with their dietitian for personalized nutrition education. The final behavior visit (month 4) involves the development of a personalized relapse prevention and maintenance plan, which again links weight loss maintenance to adolescents’ goals and values.

The TEENS+ dietary intervention was designed to yield a caloric deficit via adding low calorie, nutrient-dense foods (“Go Foods”) while remaining within a prescribed calorie range. Individual calorie and Go Food goals are provided and adjusted as needed throughout treatment [54]. Calorie goals are based on sex and height, with protocols revised in later cohorts to also include baseline weight and activity level [66], designed to yield ≥500kcal/day deficit and result in a 1-2lb/week weight loss, adjusted as needed throughout treatment. Participants are provided with a target calorie range (100kcal above and 100kcal below the target goal), intended to promote psychological flexibility, and are encouraged to remain within this range daily. Go Food goals are based on baseline food records and increased as previous goals are met, to enhance diet quality. TEENS+ focuses on adding Go Foods while reducing higher energy foods to remain within a personalized calorie range. This approach is based on prior research which found that within the context of a calorie goal, targeting an increase in healthy foods (versus a reduction in unhealthy foods) was associated with greater weight losses [67]. Moreover, in the absence of a calorie goal, interventions that target an increase in healthy foods do not yield reductions in unhealthy foods [68]. Standardized lessons provide education about energy balance, calories, macronutrients, and high-risk eating behaviors associated with obesity, coupled with evidence-based behavioral strategies to help adolescents implement changes.

The TEENS+ exercise intervention includes identifying strategies to achieve ≥1hr/day of moderate to vigorous physical activity (PA) [69]. Adolescents are provided an exercise progression based on their baseline activity levels that gradually increases the frequency and duration of PA. TEENS+ includes attending 1x/week one day of group exercise, offered at the HLC gym, with the option to also attend supervised “open gym” (arm-specific times offered) on other days. Following the transition to virtual intervention delivery, this group exercise was moved to live Zoom sessions, with audio-recorded workouts also provided. To maintain consistent contact between virtual and in-person treatment, we also added the option for adolescents to attend up to four personal training sessions, via Zoom, given the inability to continue open gym. Individual sessions also accommodate adolescents who expressed discomfort with group exercise on Zoom. Adolescents are also encouraged to exercise at the YMCA (using memberships provided) or other locations (e.g., neighborhoods, public parks) and parent/adolescent group exercise sessions are offered monthly throughout treatment. TEENS+ includes setting goals and learning strategies to achieve exercise goals with a focus on free or low-cost, realistic activities.

#### Parent interventions

Parents participate in their assigned, distinct intervention (PAC/PWL), matched on contact, led by trained, supervised lifestyle coaches (Table 1).

##### Parents as Coaches (PAC)

PAC focuses on parent skills training to support adolescents’ weight management. It includes combined nutrition education sessions with adolescents, as well as independent parent skills training group sessions, delivered while adolescents attend their own BWL groups. The TEENS+ coach is a co-facilitator of the parent sessions, to facilitate integration of content between the adolescent and parent treatments. Parents are taught authoritative parenting skills (providing structure with warmth), and strategies for applying this approach to weight management (e.g., setting limits around food kept in the house). PAC teaches positive reinforcement principles to promote behavior change, the importance of role modeling, and communication about eating and weight. Throughout, developmentally appropriate strategies to promote adolescent behavior change are emphasized. For example, parents are taught strategies for assisting their child (as needed) with logging caloric intake, highlighting the consistency of this behavior with the adolescent’s goals. This approach reduces adolescent resistance and increases autonomy, while enhancing skill development. As skills are demonstrated, parents provide positive reinforcement, and re-negotiate the level of assistance needed. In PAC, parents’ own weight management is not specifically addressed, and parent weight is not monitored. However, PAC emphasizes the importance of parents’ modeling of healthy behaviors, the value of healthy eating and exercise for the entire family (regardless of weight), and the importance of setting up the home environment to facilitate their adolescents’ successful goal attainment. PAC also emphasizes the critical need for all caregivers to use consistent feeding strategies and teaches parents strategies for communicating these concerns assertively.

Parents set specific goals each week, related to PAC treatment targets, which focus efforts on areas within their control that impact adolescent weight management behavior. Specifically, parents maintain a log to self-monitor: the number of meals planned and prepared at home for their adolescent; whether they reviewed their adolescents’ logs; responsibility for adolescent logging (parent/child/combined); and provision of Go Food and PA opportunities for their adolescent. Progress on goals is reviewed weekly, facilitating group brainstorming of strategies to overcome barriers and positively reinforce successes. Behavior coaches also deliver personalized coaching in brief individual check-ins before group, and examine the relation between parental behaviors and adolescents’ behaviors and weight change, guiding adjustments as needed to assist in goal attainment. In these ways, coaches guide parents to focus on empirically-supported strategies within their control to foster healthy adolescent behavior changes.

##### Parent Weight Loss (PWL)

In PWL, parents participate in a 16-week adult BWL program, concurrent, yet independent from their adolescents. Monthly activities foster shared engagement in BWL strategies between parents and adolescents (i.e., cooking class, monthly group exercise). Consistent with gold standard adult BWL [63, 70, 71], the intervention is grounded in an appreciation of how one’s environment (physical, social, emotional) influences weight and healthy lifestyle behaviors, and emphasizes making changes to those aspects of the environment within one’s control to support nutrient rich dietary choices and increased physical activity. The goal is to produce parent weight losses of 5-7% during the 4-month program, given reliable cardiometabolic benefits associated with weight losses of this magnitude [72]. Participants receive daily calorie and fat gram goals personalized based on baseline data and designed to yield 1-2lb/wk weight loss. Specifically, the Mifflin-St Jeor equation [66] is used to estimate resting metabolic rate. After accounting for activity level, 500kcal are subtracted to derive energy intake goals, with fat goals set as 30% of those calories. Participants are then provided with a target calorie range spanning 100 kcals below and 100 kcals above that value to promote psychological flexibility and encouraged to aim for that range consistently on a daily basis to ensure they are getting sufficient nutrients. Education about energy balance, diet quality, and calories in macronutrients provided. They are instructed to self-monitor weight, dietary intake, and PA within the context of a self-regulation framework [63] that teaches them to use this information to evaluate their behavior and make adjustments as needed to assist in goal attainment. Within an MI-consistent framework [73], parents are trained in core evidence-based behavioral strategies (e.g., goal-setting, problem solving, stimulus control) [71] to assist them in meeting their own diet and PA goals. The adapted BWL program also includes specific content focused on navigating financial, environmental, social, and emotional barriers to weight management, and implementation planning is conducted weekly in group to develop personalized plans for goal attainment.

Parents’ weight is measured weekly in a brief individual check-in prior to group, and feedback is provided in an MI-consistent manner*.* Parents also receive weekly individualized feedback from their coaches on their food diaries to affirm successes and goal progress, as well as to provide specific guidance in an autonomy supportive manner (e.g., choice of options to try to reduce sugar intake this week). Participants are encouraged to consume a heart healthy diet (low in saturated/trans-fats and rich in fruits/vegetables/whole grains). Nutrition education in PWL is consistent with that taught to adolescents with key distinctions: 1) more emphasis on reducing saturated fat as a strategy to meet calorie goals; 2) more focus on energy density; and 3) parents do not receive Go Food goals. However, to maximize use of shared strategies, PWL emphasizes consuming Go Foods within the context of diet quality and energy density, to assist in increasing satiety and adherence to calorie goals. Participants are instructed to gradually increase PA until achieving ≥250 min/wk of moderate intensity PA, with personalized progressions provided based on objectively measured activity levels at baseline. The importance of both structured PA and lifestyle activity are stressed. Typical barriers to PA are discussed, and suggestions for exercise that incorporate activities of daily living are presented. Note that PWL does not focus on how to support adolescents’ weight loss directly (although emphasizes that concurrent engagement in these behaviors should be helpful for their adolescent).

### Outcomes

All measures are psychometrically sound, appropriate for use with this population, and will be completed by masked assessors at 0, 2, 4, 8, and 12 months, unless otherwise noted. Assessments were initially conducted in person. In March 2020, fully remote protocols were implemented due to in-person research restrictions. The trial transitioned to hybrid assessment protocols once permitted by university and state guidelines, with the majority of assessments conducted in person at the HLC. Remote protocols are used as needed due to contact precautions and to allow greater flexibility for participants to enhance retention. Surveys are completed online via the secure REDCap platform [74].

#### Adolescent measures

##### Anthropometrics

At all timepoints, trained staff measure height and weight (after a 12hr fast) to the nearest 0.1cm and 0.1kg using a precision stadiometer and digital scale, respectively. BMI will be calculated (kg/m^2^). Remote/no-contact protocols include delivering a portable stadiometer and digital scale (calibrated against the trial assessment scale) to participants’ homes and conducting these measurements via video following detailed instructions.

##### Dietary intake

Participants are trained during orientation to complete a 3-day food record (using a detailed food record form) to track dietary intake for 2 weekdays and 1 weekend day in the week prior to their assessment (0, 4, 8, 12m). At their assessment, a dietitian interviews adolescents (parent-assisted) to review consumption, preparation, and portions (using food models). Diet will be analyzed via Nutrition Data System Software (NDSR) [75]. The primary dietary outcome is average total energy intake (kcal/d).

##### Physical activity

Accelerometers (Actigtraph GT9X Link) are worn for 1 week for each assessment period (0, 4, 8, 12 months) to assess physical activity objectively. Following wear-time validation [76], appropriate cut-points [77] will be applied to determine time spent in sedentary, light, moderate, and vigorous activities. Total time (min/wk) spent in moderate/vigorous activity and mean total daily PA energy expenditure (kcal/d) will be examined in analyses.

##### Psychosocial measures

Depressive symptoms are assessed using the Child Depression Inventory-2 [78]. The EDEQ-I (with BE instructions) [79–83] and the Loss of Control Eating Disorder Screening Questionnaire [84, 85] assess eating disorders and BE/LOC. PACE+ measures [86–88] evaluate family and friends’ social support for diet and PA. The Family Experiences Related to Food Questionnaire [89] measures perceptions of parental modeling of weight control behaviors. The Conflict Behavior Questionnaire [90] assesses parent-child relational factors. The Weight Control Strategies Scale (WCSS) [91] measures adolescents’ use of healthy weight control practices (diet, self-monitoring, and PA). The Perceived Parental Autonomy Support Scale assesses the extent to which adolescents feel autonomy support from their parents [92, 93]. Finally, the Parenting Style Dimension Questionnaire [94] measures adolescents’ perceptions of their caregivers’ parenting style.

#### Parent measures

##### Anthropometrics

Height and weight are measured after a 12-hour fast. BMI will be calculated (kg/m^2^).

##### Dietary intake

Parents complete their own 3-day food records (0, 4, 8, 12m) that are reviewed with a dietitian and analyzed via NDSR [75] using the same procedures applied to adolescents.

##### PA

Parent PA is assessed (at 0, 4, 8, 12 months) via accelerometers (GT9X Link) using procedures described above with appropriate cut-points to determine sedentary, light, moderate and vigorous PA [95].

##### Demographics

Parent education, household income, insurance status, family density and structure, and food insecurity [96] are assessed at 0, 4, 8, and 12 months. Age, gender, sex, race, and ethnicity are reported at baseline.

##### Psychosocial measures

The Patient Health Questionnaire-9 [97] assesses parent depression; the EDEQ-I [82] evaluates eating pathology [79, 98]. Parents complete the Conflict Behavior Questionnaire [90], which measures the parent/child relationship. Parent readiness to change to help their child lose weight [99] and readiness to make weight-related behavioral changes (for themselves) [100, 101] are also assessed, and stage of change will be determined for both variables [102]. Parent self-efficacy is be assessed with the Parent Efficacy for Child Healthy Weight Behavior [103, 104]. The WCSS [91] assesses parents’ use of healthy weight control practices (diet, self-monitoring, and PA) and is also used as a measure of parent role modeling. The Child Feeding Questionnaire (Adolescent version) [105] assesses parent feeding style. The Exercise Environment Questionnaire [106] evaluates the availability of exercise equipment in the home and the Home Food Inventory [107] assesses what food is available in the home. Finally, the Authoritative Parenting Index [108] measures parents’ perceptions of their parenting style.

##### Process measures

Attendance and adherence to self-monitoring, PA, calorie and Go Food goals are monitored (via logs). Interventionists complete fidelity checklists and a brief survey after each session assessing implementation barriers and perceptions about participant engagement. Parents and adolescents complete separate exit surveys at post-test (4 months), assessing: intervention likes/dislikes; perceived benefits and barriers to implementing intervention goals; overall satisfaction; and suggestions for improvement.

### Participant timeline

Recruitment windows open 3 months prior to treatment. Eligibility screening is ongoing, with enrollment and orientations occurring over a 2-month timeframe, and baseline assessments occurring in a 3-week window prior to treatment launch. There is a 3-week assessment window for all timepoints (2 weeks for the 2 months assessment). As noted above, the intervention occurs weekly, over a 16- week period.

### Sample size

We will recruit 210 parent/adolescent dyads (105 randomly assigned to each condition). This sample size was determined based on power analysis conducted to detect significant between-group differences in adolescent ∆BMI_4-12m_between PAC and PWL. Using estimates from our pilot data [27], we calculated the rate of change per month: PAC=.013; PWL=.156. The proposed primary endpoint of this trial is 12 months (an estimate of which is not available), thus we used these values and estimated M ∆BMI_4-12m_ to be .104 kg/m^2^ for PAC and 1.248 kg/m^2^ for PWL. The SD at 7m was 1.2 for PAC and 1.5 for PWL. Because there is likely greater variability at 12m, and the difference between the treatment groups might not continue to increase, we provide a range of scenarios to illustrate the impact on power both with the full sample (*N*=210 [105 per treatment group] in primary Intent-To-Treat [ITT] analyses) and allowing for 30% attrition (*N*=147 [~73 per treatment group]). We have >80% power, for *N*=210, if we assume the mean difference=0.86 (value observed at 7m) with SD≤2.2. If the mean difference=1.14 (estimated 12m difference), we have 80% power for SD≤2.9 (thus tolerating an almost doubling of the 7m SD, which is unlikely to occur), which corresponds to a small to moderate effect size (Cohen’s *d*=.39). For *N*=147, we have >80% power for a mean difference=0.86 (value seen at 7m) with SD≤1.8. If the mean difference=1.14 (estimated 12m difference), we have 80% power for SD≤2.4. Previous adolescent weight loss trials observed SD increases of 0.13 to 0.36 for ∆BMI from 4 months to 12 months (the same follow up duration in the current trial) [109]. Thus, with a sample size of 210 dyads, we are well-powered to test our study aims in both primary (ITT) and per protocol analyses (*N*=147). We used effect sizes to power this trial given lack of agreed-upon clinically significant thresholds for adolescent BMI change.

### Recruitment, screening and retention

Families are recruited through the HLC and local pediatricians. Recruitment occurs in waves, with 20-30 parent/child dyads per wave. Interested families complete eligibility screening via telephone, a secure website, or during an HLC clinic visit. Eligible families are invited to an orientation (in person initially, then conducted via Zoom starting with cohort 3) in which study procedures are detailed, eligibility confirmed, and written parental consent/child assent obtained. Consented participants receive detailed instructions for completing food logs and study surveys. At a subsequent visit, staff obtain baseline anthropometrics (after a 12-hr fast), place accelerometers, and review food logs. Randomization occurs following the return of the accelerometer. This baseline period takes place over ~3 weeks to address attrition early in the trial (a period of high attrition in obesity trials [110]), and prior to randomization, to ensure equal treatment allocation.

To enhance retention, we offer each family incentives to attend follow-up assessment visits ($25 at 2 months, $50 at 4 months, $75 at 8 months, and $100 at 12 month follow up). We selected this graduated incentive structure as retention is more difficult as time elapses, particularly in a no-treatment maintenance phase.

### Allocation and masking

The study biostatistician employs a block randomization scheme, stratified for adolescent sex and race, to ensure equal group allocation on these variables. Given highly skewed BMI data in the pilot (96% of adolescents had BMIs≥95^th^%ile) [27], we opted not to stratify on BMI, but review baseline data after each wave to ensure randomization is yielding groups balanced on BMI. Participants who consent/assent, meet eligibility criteria and complete baseline assessments are eligible for randomization and assigned to either TEENS+PAC or TEENS+PWL. Adolescents are in groups according to their parents’ assignment.

All assessments are completed by masked assessors at 0, 2, 4, 8, and 12 months, unless otherwise noted. Both interventions are implemented by trained individuals, masked to the study’s aims and hypotheses.

### Data collection and management

All staff involved in data collection are trained by the Principal Investigator (PI) and Study Coordinator and must demonstrate competence in completing the physical measurements described above. Questionnaire data are collected via REDCap, [74] a secure survey data collection system designed for this purpose and available through Virginia Commonwealth University. Participants receive the link to complete their measures online before their scheduled visit. The day of the visit, the (masked) research staff member conducting the assessment reviews participants’ responses for accuracy and completion. Any questions/concerns about data completion are addressed during the visit. If participants did not complete the measures prior to their appointment, they are completed on site. During the visit, research staff record physical measurements on a form developed for these purposes and used in our pilot work; this form is accompanied by a checklist prompting the assessor to review and complete all components of the visit in order (based on our Manual of Operations and in the form of a checklist). These data are entered into a password protected data entry and storage system, developed by our data manager, which provides programmatic protection against invalid data value entries, and provides second-party, blinded double-entry data verification to validate accuracy of data entry. All physical measurements data are double entered. Only those staff approved by the PI to access these data receive a log-in account. Access to the database requires a user name and password is maintained on a secure server behind a firewall. Hard copies of assessment data become part of participants’ unidentified charts, stored in locked cabinets in a locked storage room within Dr. Bean’s lab in the HLC. The study statistician is responsible for data cleaning, conducting error checks and preliminary analyses of all data to ensure accuracy.

Dr. Bean (PI) ensures that the trial is implemented according to the protocol, including adherence to eligibility criteria, safety protocols, and detailed manuals of operations. We closely monitor retention and report to the Data and Safety Monitoring Board (DSMB) at regular meetings. We have set alert points at drop-out rates of 25% (low alert; as this rate is nearing the estimated attrition rate; this will provide an early warning opportunity to address retention); 30% (moderate alert); 35% (high alert); and 40% (extreme alert). With early alerts to problems, action would be taken to avoid higher alert levels. If a higher alert level should arise, more drastic remedial action would be invoked.

### Fidelity monitoring

Interventionists are trained according to the Operations Manual. Sessions are audiotaped and reviewed in weekly supervision with Co-Investigators specific to each arm. Consistent with gold standard recommendations [111] implemented in our prior trials, 2 trained, independent raters with IRRs ≥.80 will review a random sample of 20% of audiotapes and complete fidelity checklists to assess protocol adherence and potential contamination.

### Data analyses

#### Overview

Analyses will be conducted with SAS v9.4 and Mplus. All study hypotheses will be tested using Intent-To-Treat (ITT) methods [112], with multiple imputation incorporating auxiliary variables used to handle missing data and all primary analyses will be performed using imputed data sets. We will also conduct additional, less conservative complete-case analyses and we will compare these results to the ITT analyses.

##### Preliminary exploration

Descriptive statistics will be generated for outcome variables and potential covariates (adolescent age, sex, race/ethnicity, and parent income and readiness to change) according to PAC/PWL. We will use descriptive statistics and graphical techniques prior to hypothesis testing. For categorical variables, we will examine frequency distributions, contingency tables, and histograms. For continuous variables, we will examine frequency distributions, stem-and-leaf plots, and box-and-whisker plots. If indicated, we will consider transformation. Between group baseline differences for outcomes and covariates will be assessed for continuous (via ANOVA) and categorical variables (via Chi-squared or Fisher Exact tests).

##### Aim 1

The primary outcome is between-group (PAC vs. PWL) differences in adolescent ΔBMI from 4m to 12m - weight loss maintenance. Specifically, both within- and between-group effects of treatment on weight loss maintenance will be compared via linear mixed model analyses. We will adjust for BMI at baseline. We will also model the effects of each intervention across time using longitudinal linear mixed model analysis across 2-, 4-, 8-, and 12-month outcomes, adjusting for baseline BMI, using PROC MIXED, which allows a variety of longitudinal covariance structures to be modeled. This procedure will allow us to conduct the appropriate contrast tests on the change in weight, and additionally we can compare within group effects across time.

To evaluate BE/LOC (indicated by endorsing at least weekly BE/LOC episodes on the baseline EDEQ) as a moderator we will conduct linear mixed model analysis. Treatment group and BE/LOC will be entered as main effects, and the interaction between BE/LOC and treatment group (using effects coding) entered into the model. There were no significant differences in weight loss maintenance based on adolescent sex or race (Black vs. White) in the pilot [27]; thus, we do not anticipate that race or sex will be moderators, although we will explore if differences emerge in the fully powered trial.

##### Aim 2

Both within- and between-treatment group effects on parental variables (% weight loss; child feeding behaviors [Child Feeding Questionnaire]; parenting style [parent and adolescent report of authoritative parenting based on the Parenting Style Dimension Questionnaire and Authoritative Parenting Index, respectively]; role-modeling [Weight Control Strategies Scale / Family Experiences Related to Food Questionnaire]; family social support for eating and exercise [PACE+], parent self-efficacy [Parent Efficacy for Child Healthy Weight Behavior]; energy intake [kcal/d]; energy expenditure [kcal/d]; PA [min/wk]); home exercise environment (Exercise Environment Questionnaire); home food environment (obesogenic food availability score from the Home Food Inventory); and adolescent perceived autonomy support from their parents (Perceived Parental Autonomy Support Scale) from 0-4 months will be compared via linear mixed model analyses. Multivariate linear regressions will examine the impact of 0–4-month changes in these variables on adolescent weight loss maintenance (ΔBMI_4-12m_).

We will evaluate whether *authoritative parenting*(as measured by parent and child report) mediates the association between treatment and weight loss maintenance. There are many approaches to mediation analysis [113]; we will employ an SEM approach using Mplus for these analyses. Mplus accommodates the evaluation of complex multivariate models of mediation (multiple mediators with multiple variable indicators) across time, and includes statistical tests of both direct and indirect (mediated) effects. The most basic mediated model will include paths from treatment group to authoritative parenting, from authoritative parenting to weight loss maintenance, and from treatment group to weight loss maintenance. We will also examine other theoretically and empirically supported mediators using similar methods (e.g., parent % weight loss, parent self-monitoring, home food environment, child feeding practices, adolescent perceived autonomy support) to enhance understanding of parents’ impact on adolescent weight loss maintenance in BWL treatment.

To evaluate whether BE/LOC affects the magnitude of authoritative parenting on weight loss maintenance, we will apply a moderated mediation model [114]. In this model, we anticipate that the effect of treatment on authoritative parenting and/or the effect of authoritative parenting on weight loss maintenance will be moderated by BE/LOC. For this analysis, we will adapt the SEM mediator model discussed above to a moderated mediation model using the multiplicative regression approach.

##### Secondary aim

We will apply linear mixed models, as for Aim 1, to explore within- and between-group changes in adolescent energy intake (kcal/d); energy expenditure (kcal/d); and PA (min/wk) from 4-12m.

##### Exploratory aim

Given the dyadic nature of the data, we will explore how parent and adolescent concordance of sex, weight loss, behavioral changes (self-monitoring, dietary intake, physical activity) impacts adolescent weight loss maintenance. We will also explore how the concordance of parent- and adolescent- perceived parenting style and how adolescents’ perception of the non-participating parents’ parenting style (and whether it differs from that of the participating parent) impacts adolescent weight loss maintenance. Specifically, to evaluate concordance, we will group dyads based on differences between the adolescent and the parent on the categorical measures. For concordance of continuous measures, we will first calculate difference scores, subtracting the parent’s score from the adolescent’s score. These scores will be used to create a trichotomous grouping variable distinguishing the dyads by agreement: adolescent above (positive values indicate adolescent scores are greater than parent scores), parent above (negative values indicate parent scores are greater than adolescent scores), and equal. It is highly unlikely that we will have perfect agreement between adolescents and parents on continuous measures, so the equal group will be defined as ±0.5 standard deviations around a difference score of 0. These groups will then be used in linear mixed models. Any measures significantly associated with adolescent weight loss maintenance will be evaluated as potential moderators of associations between treatment group and adolescent weight loss. Although our primary focus is on adolescent weight loss maintenance, the data collected in this study will facilitate additional exploration of dyadic methods for other outcomes, such as the behavioral changes of dietary intake and physical activity, which may inform treatment effectiveness.

### Data monitoring

Dr. Edmond Wickham (Investigator and a board-certified physician in pediatric, internal, and obesity medicine) serves as the internal medical and safety advisor. Dr. Bean (a licensed clinical psychologist) oversees psychological safety (including self-harm, suspected or confirmed child abuse/neglect, or psychopathology) of participants. Any medical or psychological safety concerns that arise are prioritized over intervention participation. As such, intervention and/or assessments are suspended as appropriate until the safety concerns are resolved. Stopping rules for suspending intervention participation are based on identification of serious psychological distress (e.g., clinically significant disordered eating, significant suicidal ideation or self-harm) or child abuse/neglect. A detailed protocol specifies the conditions that must be met before participants can resume participation in the trial if these concerns are identified (e.g., written clearance after an evaluation by a non-study psychologist; Child Protective Service investigation). A similar series of stopping or suspend rules (and associated protocols) addresses medical safety concerns (e.g., injuries or cardiac health problems). Any intervention suspension is reported to the DSMB. If additional mental or physical health concerns emerge during the course of the intervention, Dr. Bean provides appropriate referrals. The study team maintains a current list of providers for referrals, which is continually updated. If desired, the program coordinator can facilitate scheduling an appointment if the participant wishes to see a VCUHS or Children’s Hospital of Richmond provider.

An independent DSMB is responsible for monitoring the safety of the data obtained in this study. The DSMB is comprised of a physician, health psychologist, and biostatistician. Formal DSMB meetings occur twice a year, with more frequent meetings on an as-needed basis.

A record of adverse events is retained in a study binder for review by the study team, and the independent DSMB. All adverse events are initially evaluated by the PI (Bean) and medical investigator (Wickham). Those events classified as moderate or severe are reported to DSMB Committee Chair for review within 24 hours. The entire DSMB meets to review all serious adverse events, should they occur. In addition, all serious adverse events, both anticipated and unanticipated, are reported to the IRB within 48 hours (and to the NIH within a timeframe consistent with the policy of the institute). Additionally, a summary of all adverse events is submitted to the VCU IRB annually as part of the protocol’s continuing review.

### Summary

Rates of adolescent obesity are high and, if untreated, are likely to persist into adulthood. Adolescence is the last opportunity for family-based intervention, yet outcomes of adolescent BWL trials are modest. The need to include parents in treatment is evident, but the optimal approach to doing so remains unclear. This rigorous investigation uses scientifically sound methods within an RCT, stratified on key variables, contributing to high confidence in the quality and unbiased nature of the findings. This team is implementing state of the art BWL treatment and focuses on adolescent weight loss *maintenance*, using identical adolescent treatments and *distinct* parent interventions, facilitating isolation of the effects of the parent manipulation. Lastly, this trial uses objective, validated assessments, conducted by masked assessors, with a gold standard fidelity plan, and is well-powered to test the primary outcome, as well as mechanisms of action and proposed moderator analyses. Consequently, results have the potential to transform the standard of care for family-based treatment of adolescent obesity and advance science in this investigative area. Future directions include investigation of an approach that combines parent skills training with parent weight loss and examination of maintenance effects beyond 12 months. Future research will also investigate a family-based approach to weight loss and BE/LOC, with a specific focus on strategies to maintain treatment gains in both domains. Results of these studies will inform the development of tailored adolescent treatments, ultimately informing clinical practice guidelines related to the role of parents in adolescent obesity treatment, and guiding dissemination into community-based settings.

## Abbreviations

PAC: Parents as Coaches
PWL: Parent Weight Loss
PA: Physical Activity
BMI: Body mass index
RCT: Randomized Clinical Trial
BWL: Behavioral Weight Loss
BE/LOC: Binge Eating/Loss of Control
